# Analysis of risk factors for the sigmoid stoma complications in patients after abdominoperineal resection surgery: An observational study

**DOI:** 10.1097/MD.0000000000038751

**Published:** 2024-06-28

**Authors:** Quan Lv, Ye Yuan, Zheng Xiang

**Affiliations:** a Department of Gastrointestinal Surgery, The First Affiliated Hospital of Chongqing Medical University, Chongqing, China; b Chongqing Key Laboratory of Department of General Surgery, The First Affiliated Hospital of Chongqing Medical University, Chongqing, China.

**Keywords:** APR surgery, complications, operation time, rectal cancer, stoma

## Abstract

To analyze the risk factors for intraperitoneal sigmoid stoma complications after abdominoperineal resection (APR) surgery to guide clinical practice. Patients who were diagnosed with rectal cancer and underwent APR surgery from June 2013 to June 2021 were retrospectively enrolled. The characteristics of the stoma complication group and the no stoma complication group were compared, and univariate and multivariate logistic analyses were employed to identify risk factors for sigmoid stoma-related complications. A total of 379 patients who were diagnosed with rectal cancer and underwent APR surgery were enrolled in this study. The average age of the patients was 61.7 ± 12.1 years, and 226 (59.6%) patients were males. Patients in the short-term stoma complication group were younger (55.7 vs 62.0, *P* < .05) and had a more advanced tumor stage (*P* < .05). However, there was no significant difference between the long-term stoma complication group and the no stoma complication group. Multivariate logistic regression analysis revealed that operation time was an independent risk factor (*P* < .05, OR = 1.005, 95% CI = 1.000–1.010) for short-term stoma complications. Both the short-term and long-term stoma complication rates in our institution were low. A longer operation time was an independent risk factor for short-term stoma complications after APR surgery.

## 1. Introduction

Colorectal cancer (CRC) is the second most prevalent cancer in the world, and its incidence is increasing year by year. There are a large number of CRC patients in China because of its large population. Compared to Western countries, rectal cancer (RC), especially low rectal cancer (LRC), accounts for the majority of CRC cases in China.^[1–3]^ The treatment of CRC still relies on surgery-based comprehensive treatment.^[4–10]^ When RC is close to the anus, most patients have to undergo abdominoperineal resection (APR) surgery with a sigmoid colostomy.^[11–14]^ Although advances in surgical techniques have allowed more opportunities for anal preservation in patients with LRC, postoperative dysfunction and higher recurrence rates make the patient’s quality of life (QoL) much worse than that of patients with a stoma.^[15–19]^

Stoma-related complications often severely reduce the QoL of stoma patients after surgery and are a major concern for surgeons.^[20–26]^ It is closely related to the surgical technique, especially to the stoma approach.^[27–29]^ Currently, there are 2 main types of sigmoid colostomies: intraperitoneal and extraperitoneal. Some studies have reported a lower incidence of stoma complications with the extraperitoneal colostomy than with the intraperitoneal colostomy.^[30,31]^ However, Heiying et al^[32]^ demonstrated that there was no significant difference in the incidence of stoma complications between extraperitoneal and intraperitoneal colostomy, and even intraperitoneal colostomy had a lower incidence of long-term complications. Moreover, there was no previous research examining the risk factors for complications associated with sigmoid stomas after APR surgery. Therefore, because intraperitoneal sigmoid colostomy is the more common surgical option, we performed this study to analyze the risk factors for intraperitoneal sigmoid stoma complications after APR surgery to guide clinical practice.

## 2. Methods

### 2.1. Patients

Patients who were diagnosed with RC and underwent APR surgery at the First Affiliated Hospital of Chongqing Medical University from June 2013 to June 2021 were retrospectively enrolled.

### 2.2. Inclusion and exclusion criteria

**Figure 1.**
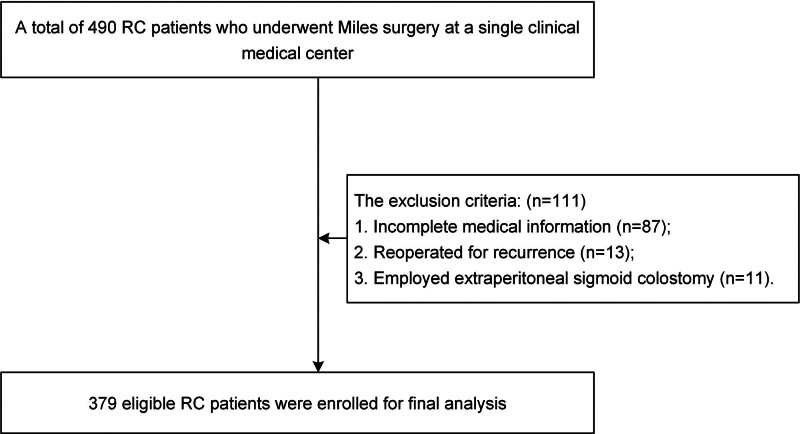
Flow chart of patient selection.

Patients who were over 18 years old and received APR surgery were included in this study (n = 490). The exclusion criteria were as follows: incomplete medical information (n = 87); recurrence resulting in a second operation (n = 13); and extraperitoneal sigmoid colostomy (n = 11). Finally, there were 379 patients enrolled in this study (Fig. 1).

### 2.3. Surgical procedure of sigmoid colostomy

**Figure 2.**
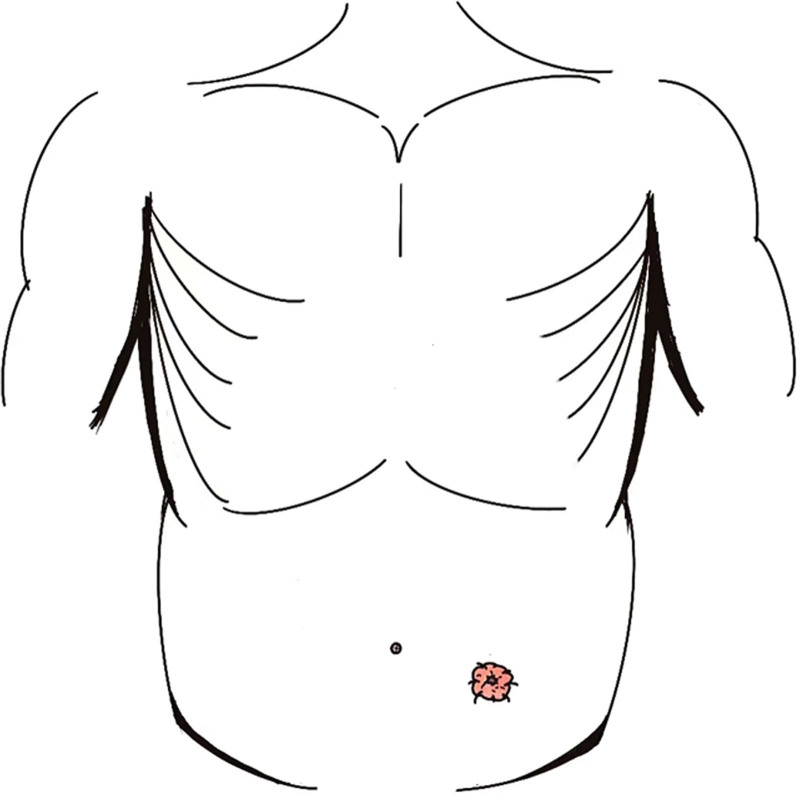
Intraperitoneal sigmoid stoma after APR surgery. APR = abdominoperineal resection.

A circular incision of 2.5 cm in diameter was first made in the medial superior third of the line between the navel and the anterior superior iliac crest, and the skin and subcutaneous fat were removed (Fig. 2). The anterior rectus abdominis sheath was transected, and the rectus abdominis muscle was bluntly separated. The posterior rectus abdominis sheath and peritoneum were then transected. The sigmoid stump was dragged through the tunnel to approximately 2 cm above the skin surface. The intestinal wall was sutured to the peritoneum and the anterior rectus abdominis sheath. Finally, the intestinal wall and skin were intermittently sutured after the stump was opened and disinfected.

### 2.4. Data collection

Baseline information, short-term stoma complications, and long-term stoma complications were collected from electronic medical records for analysis. The baseline information included age, sex, body mass index (BMI), smoking, drinking, hypertension, type 2 diabetes mellitus (T2DM), surgery method, surgical history, tumor size, TNM stage, operation time, and intraoperative blood loss. Short-term stoma complications (occurring within 1 month) included stoma bleeding, stoma prolapse, stoma necrosis, and stoma stenosis. Long-term stoma complications (occurring after 1 month) included parastomal hernia, skin inflammation around the stoma, stoma stenosis, and stoma prolapse.

### 2.5. Statistical analysis

For continuous variables, we performed normal distribution analyses. Variables that conformed to normal distribution were expressed as mean ± SD and subjected to the independent samples *t* test; variables that did not conform to normal distribution were expressed as median with interquartile range (IQR) and subjected to the Mann–Whitney *U* test. Frequency variables were expressed as n (%), and the chi-square test or Fisher exact test was used. A univariate logistic regression analysis was conducted to identify potential risk factors for stoma-related complications. Factors with a *P* value < .05 were included in the final multivariate logistic regression for identifying independent risk factors. The data were analyzed using SPSS (version 22.0) statistical software. A bilateral *P* value of < .05 was considered statistically significant.

## 3. Results

### 3.1. Baseline characteristics of patients

A total of 379 patients who were diagnosed with RC and underwent APR surgery were enrolled in this study. The average age of the patients was 61.7 ± 12.1 years, and 226 (59.6%) patients were males. The incidence of short-term stoma complications was 17 (4.5%), while that of long-term stoma complications was (19) 5.0%. The most frequent short-term stoma complication was stoma bleeding (3.2%). Skin inflammation around the stoma was the most common long-term stoma complication (2.4%). Other clinical characteristics are summarized in Table 1.

**Table 1:** Clinical characteristics.

Characteristics	No. 379
Age, yr	61.7 ± 12.1
Sex	
Male	226 (59.6%)
Female	153 (40.4%)
BMI, kg/m^2^	22.7 ± 3.3
Smoking	140 (36.9%)
Drinking	112 (29.6%)
Hypertension	87 (23.0%)
T2DM	40 (10.6%)
Open surgery	19 (5.0%)
Surgical history	90 (23.7%)
Tumor size	
<5 cm	294 (77.6%)
≥5 cm	85 (22.4%)
TNM stage	
I	114 (30.1%)
II	136 (35.9%)
III	129 (34.0%)
Operation time (median, IQR; min)	245 (100)
Blood loss (median, IQR; mL)	100 (120)
Short-term stoma complications	17 (4.5%)
Stoma bleeding	12 (3.2%)
Stoma prolapse	1 (0.3%)
Stoma necrosis	1 (0.3%)
Stoma stenosis	3 (0.8%)
Long-term stoma complications	19 (5.0%)
Parastomal hernia	4 (1.1%)
Skin inflammation around the stoma	9 (2.4%)
Stoma stenosis	3 (0.8%)
Stoma prolapse	3 (0.8%)

Variables are expressed as the mean ± SD or median with IQR, n (%).

BMI = body mass index, IQR = interquartile range, T2DM = type 2 diabetes mellitus.

### 3.2. Comparison between the stoma complication group and the no stoma complication group

Patients in the short-term stoma complication group were younger (55.7 vs 62.0, *P* < .05) and had a more advanced tumor stage (*P* < .05) (Table 2). However, there was no significant difference between the long-term complication group and the no stoma complication group (Table 3). Therefore, we only performed univariate and multivariate logistic analyses of short-term stoma complications.

**Table 2:** Comparison between the short-term stoma complication group and the no stoma complication group.

Characteristics	Stoma complication (n = 17)	No stoma complication (n = 343)	*P* value
Age, yr	55.7 ± 10.8	62.0 ± 12.2	.038*
Sex			
Male	11 (64.7%)	203 (59.2%)	.651
Female	6 (35.1%)	140 (40.8%)	
BMI, kg/m^2^	22.1 ± 3.0	22.7 ± 3.3	.508
Smoking	8 (47.1%)	125 (36.4%)	.376
Drinking	5 (29.4%)	100 (29.2%)	1.000
Hypertension	4 (23.5%)	79 (23.0%)	1.000
T2DM	1 (5.9%)	38 (11.1%)	1.000
Surgical history	2 (11.8%)	84 (24.5%)	.381
Open surgery	1 (5.9%)	16 (4.7%)	.569
TNM stage			
I	2 (11.8%)	106 (30.9%)	.035*
II	11 (64.7%)	118 (34.4%)	
III	4 (23.5%)	119 (34.7%)	
Tumor size			
<5cm	16 (94.1%)	265 (77.3%)	.135
≥5cm	1 (5.9%)	78 (22.7%)	
Operation time (median, IQR; min)	247 (65)	245 (100)	.109
Blood loss (median, IQR; mL)	100 (125)	100 (100)	.973

Variables are expressed as the mean ± SD or median with IQR, n (%).

BMI = body mass index, IQR = interquartile range, T2DM = type 2 diabetes mellitus.

* *P* value < .05.

**Table 3:** Comparison between the long-term stoma complication group and the no stoma complication group.

Characteristics	Stoma complication (n = 19)	No stoma complication (n = 343)	*P* value
Age, yr	61.6 ± 10.4	62.0 ± 12.2	.884
Sex			
Male	12 (63.2%)	203 (59.2%)	.731
Female	7 (36.8%)	140 (40.8%)	
BMI, kg/m^2^	23.5 ± 2.9	22.7 ± 3.3	.274
Smoking	7 (36.8%)	125 (36.4%)	.972
Drinking	7 (36.8%)	100 (29.2%)	.475
Hypertension	4 (21.1%)	79 (23.0%)	1.000
T2DM	1 (5.3%)	38 (11.1%)	.707
Surgical history	4 (21.1%)	84 (24.5%)	1.000
Open surgery	2 (10.5%)	16 (4.7%)	.242
TNM stage			
I	6 (31.6%)	106 (30.9%)	.959
II	7 (36.8%)	118 (34.4%)	
III	6 (31.6%)	119 (34.7%)	
Tumor size			
<5 cm	13 (68.4%)	265 (77.3%)	.403
≥5 cm	6 (31.6%)	78 (22.7%)	
Operation time (median, IQR; min)	265 (127)	245 (100)	.622
Blood loss (median, IQR; mL)	100 (100)	100 (100)	.654

Variables are expressed as the mean ± SD or median with IQR, n (%).

BMI = body mass index, IQR = interquartile range, T2DM = type 2 diabetes mellitus.

*P value < .05.

### 3.3. Univariate and multivariate logistic analyses of short-term stoma complications

Univariate logistic analysis was conducted to identify potential factors for short-term sigmoid stoma-related complications, and we found that age (*P* < .05, OR = 0.961, 95% CI = 0.925–0.998) and operation time (*P* < .05, OR = 1.005, 95% CI = 1.001–1.010) were potential risk factors for short-term stoma-related complications. Furthermore, in the multivariate analysis, operation time was an independent risk factor (*P* < .05, OR = 1.005, 95% CI = 1.000–1.010) (Table 4).

**Table 4:** Univariate and multivariate logistic regression analysis of the short-term stoma complications.

Risk factors	Univariate analysis		Multivariate analysis	
	OR (95% CI)	*P* value	OR (95% CI)	*P* value
Age, yr	0.961 (0.925–0.998)	0.039*	0.962 (0.925–1.001)	.056
Sex (female/male)	0.798 (0.289–2.205)	0.663		
BMI, kg/m^2^	0.945 (0.810–1.102)	0.469		
Hypertension (yes/no)	1.034 (0.328–3.257)	0.954		
T2DM (yes/no)	0.518 (0.067–4.011)	0.528		
Surgical methods (open/laparoscopic)	1.194 (0.150–9.514)	0.867		
Tumor stage (III/II/I)	1.137 (0.616–2.098)	0.681		
Smoking (yes/no)	1.549 (0.584–4.111)	0.380		
Drinking (yes/no)	0.993 (0.341–2.887)	0.867		
Surgical history (yes/no)	0.415 (0.093–1.851)	0.249		
Tumor size (≥5/<5), cm	0.207 (0.027–1.583)	0.129		
Blood loss, mL	0.999 (0.995–1.003)	0.574		
Operation time, min	1.005 (1.001–1.010)	0.030*	1.005 (1.000–1.010)	.046*

* *P* value < .05.

BMI = body mass index, CI = confidence interval, T2DM = type 2 diabetes mellitus, OR = Odds ratio.

## 4. Discussion

A total of 379 patients were enrolled in this study. Short-term stoma complications, including stoma bleeding, prolapse, necrosis, and stenosis, occurred in 4.5% of patients. Long-term stoma complications, including encompassing parastomal hernia, skin inflammation around the stoma, stoma stenosis, and stoma prolapse, occurred in 5.0% of patients. Patients in the short-term stoma complication group were younger and had a more advanced tumor stage than those in the no stoma complication group. Furthermore, multivariate logistic regression analysis suggested that a longer operation time was a risk factor for short-term stoma complications.

CRC has a high incidence among cancers worldwide. LRC accounts for most CRC cases in China.^[1–3]^ LRC often requires APR surgery to ensure sufficient margins to reduce the recurrence rate after surgery.^[11–14]^ It has been reported that when the local infiltration depth is shallow, intersphincteric resection (ISR) can be performed to achieve anal preservation despite the location being close to the anus.^[15–19]^ However, the functional impairment and higher recurrence rate of patients after ISR made the QoL low or even inferior to that of stoma patients who underwent APR surgery.

Stoma patients generally had low QoL, especially when stoma-related complications occurred.^[20–26]^ Stoma-related complications are closely related to the surgical technique.^[27–29]^ Currently, there are 2 main types of sigmoid stomas: intraperitoneal and extraperitoneal. Dong et al^[30]^ reported that the extraperitoneal colostomy could reduce the occurrence of stoma-related complications and shorten the length of the postoperative hospital stay, potentially improving patients’ QoL. Wang et al^[31]^ also reported that extraperitoneal ostomy had fewer complications. However, Heiying et al^[32]^ demonstrated that there was no significant difference in the incidence of stoma-related complications between extraperitoneal and intraperitoneal colostomy, and even the intraperitoneal colostomy group had a lower incidence of long-term complications (Table 5). It was controversial as to which surgical approach was better. Clinically, intraperitoneal colostomy was still more predominant. Moreover, there was no previous research examining the risk factors for complications associated with sigmoid stoma after APR surgery. Therefore, we performed this study to analyze the risk factors for intraperitoneal sigmoid stoma complications after APR surgery to guide clinical practice.

**Table 5:** The difference between extraperitoneal and intraperitoneal colostomy group reported in previous studies.

Author	Year	Country	Sample size	EC	IC	Outcomes
Dong et al^[30]^	2012	China	128	66	62	In conclusion, extraperitoneal colostomy without damaging the muscle layer of the abdominal wall can simplify the stoma operation, save surgery time, reduce damage to the structure of the abdominal wall, reduce the occurrence of stoma-related complications and shorten the length of postoperative hospital stay, potentially improving patients’ quality of life
Heiying et al^[32]^	2014	China	36	18	18	In conclusion, the laparoscopic extraperitoneal ostomy is an easy and safe procedure. It did not increase complications following the operation. The long-term complications were lower in the extraperitoneal ostomy group
Wang et al^[31]^	2018	China	231	108	123	Extraperitoneal sigmoidostomy is associated with shorter operative duration and postoperative hospitalization and has fewer complications and better outcome for abdominoperineal resection of rectal cancer, and patients also had less psychological problems

EC = extraperitoneal colostomy; IC = intraperitoneal colostomy.

The incidence of complications with an intraperitoneal stoma in our study was 9.5%. This was much lower than the incidence of both intraperitoneal and transrectal extraperitoneal stoma complications reported in previous literature. This might be attributed to better health education and care after the stoma was formed. Our hospital has a dedicated stoma team for education and stoma bag replacement and a dedicated stoma clinic for postdischarge patients.^[33–38]^

In this study, we found that the group with short-term stoma complications was younger and had a more advanced tumor stage. Tumors generally occur in elderly individuals, and some studies have reported that younger cancer patients tend to have more malignant tumors.^[39–41]^ The higher malignancy and more advanced stage usually indicate stronger invasiveness, which could cause lymphatic reflux disorders in the intestinal wall and lead to bowel wall edema. Then, the edematous intestinal wall might contribute to stoma bleeding, prolapse, necrosis, and stenosis. Multifactorial logistic regression analysis further confirmed that a longer operation time was an independent risk factor for short-term complications. As the longer the procedure takes, the longer the anesthesia, the more difficult the procedure was and the more complications it may lead to after the procedure.

For long-term stoma complications, there was no significant difference in characteristics between the long-term stoma complication group and the no stoma complication group. We speculated that long-term postoperative complications might be more related to surgical technique and postoperative care than to patients’ baseline information.

To our knowledge, this was the first study to examine the risk factors for post-APR intraperitoneal sigmoid stoma complications. The sample size was larger than that in previous studies, and logistic regression analysis was used for the first time to determine independent risk factors for stoma-related complications.

There were limitations in our study as well. First, this was a retrospective study with imperfect information on the included patients, and there may have been some selection bias. Second, we only explored the postoperative complications of the intraperitoneal stoma. It is still unclear whether intraperitoneal or extraperitoneal colostomy was better, and further study is warranted. Third, although the sample size was larger than in any previous related literature, it was still not large enough.

In conclusion, both the short-term and long-term stoma complication rates in our institution were low. A longer operation time was an independent risk factor for short-term stoma complications.

## Acknowledgments

The authors are grateful to all the colleagues who helped in the preparation of this article.

## Author contributions

**Data curation:** Quan Lv, Ye Yuan.

**Quality assessments:** Ye Yuan.

**Data analysis:** Quan Lv, Zheng Xiang.

**Writing – original draft:** Quan Lv.

**Funding acquisition:** Zheng Xiang.

**Writing – review & editing:** Zheng Xiang, Ye Yuan.
